# Psychological, psychiatric, and behavioral sciences measurement scales: best practice guidelines for their development and validation

**DOI:** 10.3389/fpsyg.2024.1494261

**Published:** 2025-01-23

**Authors:** Alberto Stefana, Stefano Damiani, Umberto Granziol, Umberto Provenzani, Marco Solmi, Eric A. Youngstrom, Paolo Fusar-Poli

**Affiliations:** ^1^Department of Brain and Behavioral Sciences, University of Pavia, Pavia, Italy; ^2^Department of General Psychology, University of Padua, Padua, Italy; ^3^SCIENCES Lab, Department of Psychiatry, University of Ottawa, Ottawa, ON, Canada; ^4^Department of Mental Health, The Ottawa Hospital, Ottawa, ON, Canada; ^5^Clinical Epidemiology Program, Ottawa Hospital Research Institute, University of Ottawa, Ottawa, ON, Canada; ^6^Faculty of Medicine, School of Epidemiology and Public Health, University of Ottawa, Ottawa, ON, Canada; ^7^Department of Child and Adolescent Psychiatry, Charité Universitätsmedizin, Berlin, Germany; ^8^Division of Child and Family Psychiatry, Institute for Mental and Behavioral Health Research, Nationwide Children’s Hospital, The Ohio State University, Columbus, OH, United States; ^9^Department of Psychology and Neuroscience, University of North Carolina at Chapel Hill, Chapel Hill, NC, United States; ^10^Helping Give Away Psychological Science, Chapel Hill, NC, United States; ^11^OASIS Service, South London and Maudsley NHS Foundation Trust, London, United Kingdom; ^12^Early Psychosis: Interventions and Clinical-detection (EPIC) Lab, Department of Psychosis Studies, Institute of Psychiatry, Psychology and Neuroscience, King’s College London, London, United Kingdom

**Keywords:** scale development, scale validation, evidence-based assessment, psychological measurement, psychiatric measurement

## Abstract

Psychiatric, psychological, and behavioral sciences scales provide quantitative representations of phenomena such as emotions, beliefs, functioning, and social role perceptions. Methodologists and researchers have criticized current scale development practices, emphasizing that inaccurate measurements can derail theory development and clinical decisions, thereby impeding progress in mental health research and practice. These shortcomings often stem from a lack of understanding of appropriate scale development techniques. This article presents a guide to scope, organize, and clarify the process of scale development and validation for psychological and psychiatric use by integrating current methodological literature with the authors’ real-world experience. The process is divided into five phases comprising 18 steps. In the Preliminary Phase, the need for a new scale is assessed, including a review of existing measures. In the Item Development Phase, the construct is defined, and an initial pool of items is generated, incorporating literature reviews, expert feedback, and target population evaluation to ensure item relevance and clarity. During the Scale Construction Phase, the scale is finalized through the administration of surveys to a large sample, followed by parallel analysis, exploratory factor, and item descriptive statistics to identify functional items. In the Scale Evaluation Phase, the dimensionality, reliability, and validity of the scale are rigorously tested using both classical and modern psychometric techniques. Finally, in the Finalization Phase, the optimal item sequence is decided, and a comprehensive inventory manual is prepared. In sum, this structured approach provides researchers and clinicians with a comprehensive methodology for developing reliable, valid, and user-friendly psychological, psychiatric, and behavioral sciences measurement scales.

## Introduction

Psychiatric and psychological scales provide quantitative representations of Phenomena such as beliefs, motivations, expectations, emotions, functioning, and social role perceptions that cannot be directly measured but play a crucial role in shaping social, emotional, and mental health disorders. In clinical settings, efficient assessment is essential because it leads to a better and more accurate diagnosis (Jenkins et al., 2012; Youngstrom et al., 2018), better psychological/psychopharmacological treatment matching (Durosini and Aschieri, 2021; Martinez-Aran and Vieta, 2022; Youngstrom et al., 2017), increased patient engagement (Kealy et al., 2019; Lambert et al., 1998), and improved outcomes (Wright et al., 2022; Youngstrom and Van Meter, 2016).

Developing a reliable, valid, and versatile scale is a complex process that demands systematic and thorough methodological (DeVellis and Thorpe, 2022; Lane, 2015), psychometric (Irwing et al., 2018; Swan et al., 2023), and ethical (Leach and Oakland, 2007) procedures. Inaccurate measurements can derail theory development and clinical decisions, impeding progress in mental health research and practice. Methodologists and researchers have voiced criticism regarding inadequacies in development practices, arguing that seriously flawed measures have been published even in high-impact journals (Boateng et al., 2018; Kline, 2023). These shortfalls often arise from a lack of understanding of appropriate scale development techniques and reporting procedures (Carpenter, 2018).

This article aims to present a guide to scope, organize, and clarify the process of scale development for psychological and psychiatric use by integrating up-to-date methodological literature with the authors’ real-world experience. The scoping aspect gathers multiple perspectives and recommendations about best practices, which we then organize into a sequence which is not rigid, but which would be an example of an efficient order of operations for a program of scale development research.

## Core steps in scale development and validation

**Figure 1:**
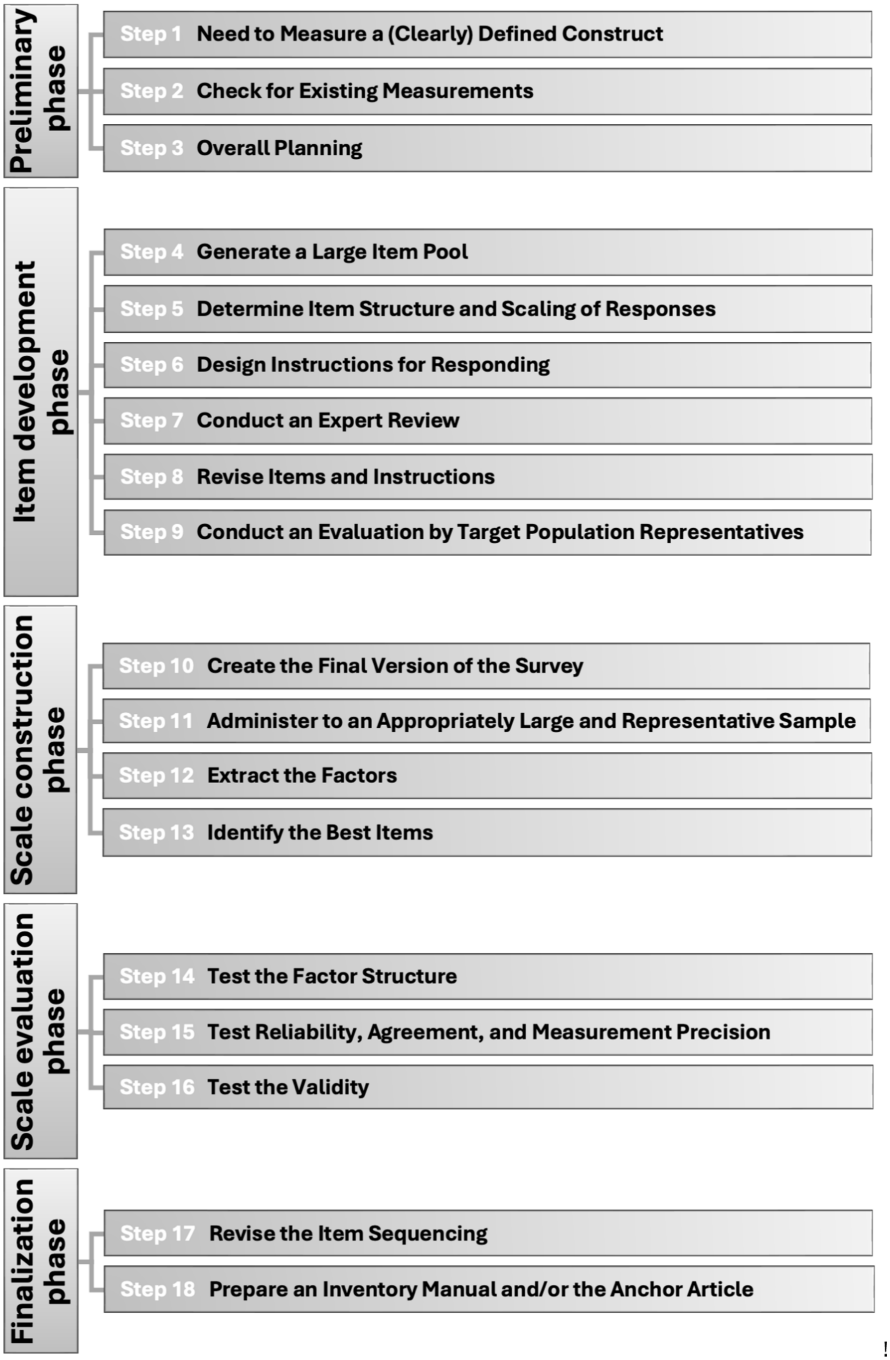
Overview of phases and their steps for scale development and validation.

A rigorous scale development process entails a series of fundamental steps that can be revisited iteratively throughout development (American Educational Research Association, American Psychological Association, and National Council on Measurement in Education, 2014; Boateng et al., 2018; Carpenter, 2018; Clark and Watson, 2019; DeVellis and Thorpe, 2022; Irwing et al., 2018; Kyriazos and Stalikas, 2018; McCoach et al., 2013; Streiner et al., 2015; Swan et al., 2023; Wilson, 2023; Zickar, 2020). We have identified five phases that encompass eighteen steps (see Table 1; Figure 1), which will be outlined in detail in the subsequent sections. Although an in-depth analysis of the technical psychometric aspects is beyond the scope of this article, we will provide specific references for the readers interested in these details.

**Table 1:** Key phases and steps of scale development and validation.

Steps	Phases and respective activities	References
	**Preliminary phase** [A]	
Step 1	**Need to measure a (clearly) defined construct** A.1.0. Identify a genuine need within clinical or research practice. A.1.1. Conduct a thorough literature review. [V] A.1.2. Define the construct(s) and identify any potential dimensions. [V] A.1.3. Ascertain the dimensional nature of the construct and determine the appropriate level of measurement. [V] A.1.4. Formulate a theoretically or empirically grounded hypothesized model of the construct. [V] A.1.5. Formulate an explicit operational definition for the construct. [V]	Borsboom (2009), Carpenter (2018), Clark and Watson (2019), DeVellis and Thorpe (2022), Edwards and Bagozzi (2000), Haynes et al. (1995), Irwing and Hughes (2018), McCoach et al. (2013), and Streiner et al. (2015)
Step 2	**Check for existing measurements** A.2.0. Verify the availability of suitable existing measurements. If no appropriate tools are available: A.2.1. Provide a rationale for the development of a new instrument. If established tools are in use: A.2.2. Explain how the new instrument offers theoretical or empirical improvements over current measures.	
Step 3	**Overall planning** A.3.0. Assemble the test development team and define individual roles and responsibilities. A.3.1. Define the purpose and structure of the test. A.3.2. Establish a detailed timeline.	Roid (2016)
	**Item development phase** [B]	
Step 4	**Generate a large item pool** B.4.0. Create a substantial pool of potential items, three to four times the size of the intended final scale. [R & V] B.4.1. Involve target population representatives (usually, patients) to explore their lived experience. [V] B.4.2. Adopt a mixed-methods strategy, combining inductive insights from empirical data with deductive reasoning from literature and existing scales. [R & V] B.4.3. Engage both quantitative methods for analyzing numerical data and qualitative methods for understanding non-numeric data. [R & V] B.4.4. Maintain clarity and brevity in item language and be cautious with the mixing of positively and negatively worded items. [R] These sub-steps are often undertaken in a simultaneous and iterative process.	Clark and Watson (2019), Dimitrov (2012), Food and Drug Administration (2018, 2020), Götz et al. (2023), Haladyna and Rodriguez (2013), Hinkin (2005), McCoach et al. (2013), McKenna (2011), Messick (1995), Netemeyer et al. (2003), and Ricci et al. (2019)
Step 5	**Determine item structure and scaling of responses** B.5.0. Select the appropriate level of abstraction for item structures. [V] B.5.1. Opt for a specific type of response format, avoiding bipolar items (e.g., “Agree” vs. “Disagree”) when possible. [R] B.5.2. Establish the number of response categories or the scale’s length. [R] B.5.3. Decide whether to specify the item time frame or leave it implicit. [R]	Barnette (2000), DeVellis and Thorpe (2022), Gadermann et al. (2012), Krosnick (2018), Preston and Colman (2000), Schuman and Presser (1996), Sliter and Zickar (2014), and Streiner et al. (2015)
Step 6	**Design instructions for responding** 6.0. Create clear instructions that are easy to understand by the target population. [R]	DeVellis and Thorpe (2022), and McCoach et al. (2013)
Step 7	**Conduct an expert review** B.7.0. Review the initial item set for relevance, validity, and clarity of the items content, as well as the appropriateness of the response scale and the instructions. This should be conducted by a panel of 3–10 experts, including both methodologists and content experts (both researchers and clinicians). [R & V]	DeVellis and Thorpe (2022), McCoach et al. (2013), Ruel et al. (2016), Streiner et al. (2015), and Willis (2005)
Step 8	**Revise items and instructions** B.8.0. Revise instructions and items based on expert feedback. [R & V]	DeVellis and Thorpe (2022), McCoach et al. (2013), and Streiner et al. (2015)
Step 9	**Conduct an evaluation by target population representatives** B.9.0. Assess items for relevance and true representation of the experiences of the target population through cognitive interviewing, while also detecting potential ambiguities within the assessment items. Involve 5–15 representatives of the target population. [R & V]	Beatty and Willis (2007), Collins (2003), DeVellis and Thorpe (2022), Foddy (1993), Peterson et al. (2017), and Streiner et al. (2015)
	**Scale construction phase** [C]	
Step 10	**Create the final version of the survey** C.10.0. Refine scale instructions and items based on feedback from target population representatives. [R & V] C.10.1. Add a concise set of sociodemographic and clinical questions. [V] C.10.2. Consider including items that evaluate the construct validity of the scale. [V] C.10.3. Format the surveys to be professional in appearance, visually appealing, and user-friendly for readability. [R]	Dillman et al. (2014), Lam et al. (2002), McCoach et al. (2013), Şahin (2021), and Schell and Oswald (2013)
Step 11	**Administer to an appropriately large and representative sample** C.11.0. Estimate the sample size necessary for reliable factor analysis. [R] C.11.1. Collect baseline data from an initial sample for scale development. [R & V] C.11.2. When possible, obtain from an independent sample or from the original sample at a subsequent time, to validate the scale. [R & V]	Comrey and Lee (2013), DeVellis and Thorpe (2022), Dillman et al. (2014), Gosling and Mason (2015), Gorsuch (2014), Kline (2023), Kyriazos (2018), Myors and Murphy (2023), Osborne (2014), Tabachnick et al. (2019), and Whittaker and Schumacker (2022)
Step 12	**Extract the factors** C.12.0. Confirm data suitability with pre-factor analysis tests. [R & V] C.12.1. Conduct parallel analysis using multiple methods. [R & V] C.12.2. Determine the number of factors to retain by integrating parallel analysis, scree test results, and theoretical considerations. [R & V] C.12.3. Execute exploratory factor analysis (EFA) and/or exploratory graph analysis (EGA). [R & V] Revisit initial steps of scale development if EFA/EGA results diverge from theoretical expectations.	Carpenter (2018), Courville and Thompson (2001), DeVellis and Thorpe (2022), Henson and Roberts (2006), Irwing et al. (2018), McCoach et al. (2013), and Thompson and Daniel (1996)
Step 13	**Identify the best items** C.13.0. Detect and evaluate outliers. C.13.1. Assess the assumption of multivariate normality. C.13.2. Identify functional items by using both traditional (test-level) and modern (item-level) test theories, selecting from techniques like item difficulty and item discrimination indexes, inter-item and item-total correlations, tailored to your scale’s particular characteristics. [R & V] C.13.3. Eliminate items that have excessive missing data, problematic cross-loadings, or poor factor loadings, as well as items with limited theoretical convergence or those that negatively impact scale reliability and discriminative capacity. [R & V] C.13.4. Balance the benefits of removing items to enhance psychometric properties against the potential loss of meaningful content and theoretical coherence. [R & V] C.13.5. Aim for an average interitem correlation between 0.30 and 0.50 to ensure scale homogeneity. [R] C.13.6. Ensure that each subscale contains a minimum of three items to capture the construct’s dimensions adequately. [R & V]	Carpenter (2018), Clark and Watson (2019), Crocker and Algina (2008), DeVellis and Thorpe (2022), 2022, Dimitrov (2012), Hair et al. (2022), McCoach et al. (2013), Parent (2013), Raykov and Marcoulides (2011), Streiner et al. (2015), and Worthington and Whittaker (2006)
	**Scale evaluation phase** [D]	
Step 14	**Test the factor structure** D.14.0. Conduct a confirmatory factor analysis. [R & V] D.14.1. Conduct multiple-group confirmatory factor analysis. [R & V] If full scalar invariance is not achieved, D.14.2. Employ the alignment method. [R & V]	Asparouhov and Muthén (2014), Bandalos and Finney (2018), Brown (2015), Chen (2007), Hoyle (2023), Hu and Bentler (1999), Kline (2023), Morin et al. (2020), Prokofieva et al. (2023), Putnick and Bornstein (2016), Reise et al. (2023), and Rodriguez et al. (2016)
Step 15	**Test reliability, agreement, and measurement precision** D.15.0. Evaluate internal consistency with McDonald’s omega, Cronbach’s alpha (if appropriate), and average interitem correlation. [R] D.15.1. Assess test–retest reliability using data collected at multiple time points, if any. [R] D.15.2. Assess agreement and measurement precision. [R]	Bland and Altman (1986), de Vet et al. (2017), Hernaez (2015), Revelle and Condon (2019), Streiner et al. (2015), and Swan et al. (2023)
Step 16	**Test the validity** D.16.0. Assess content validity. [V] D.16.1. Assess criterion-related validity (evaluated through both predictive and concurrent validity). [V] D.16.2. Assess convergent validity. [V] D.16.3. Assess discriminant validity. [V]	Almanasreh et al. (2019), DeVellis and Thorpe (2022), McDonald (1999), Raykov and Marcoulides (2011), Strauss and Smith (2009), Streiner et al. (2015), and Westen and Rosenthal (2003)
	**Finalization phase** [E]	
Step 17	**Revise the item sequencing** E.17.0. Determine the optimal sequence of items, considering the scale’s structure and the constructs (and their dimensions) it measures. [R & V] E.17.1. Perform preliminary testing with a representative sample, if feasible, to refine item sequencing. [R & V]	Sudman et al. (1996) and Wilson (2023)
Step 18	**Prepare an inventory manual and/or the anchor article** E.18.0. Prepare a concise yet comprehensive report. E.18.1. Consider making the report accessible as an inventory manual via open-access platforms. E.18.2. Publish the report as a peer-reviewed journal article. E.18.3. Submit it for classification by relevant regulatory bodies and ensure it is indexed in test repositories. E.18.4. Periodically revise the inventory to account for advances in theory, changes in the construct being measured, or the presence of outdated items.	DeVellis and Thorpe (2022), McCoach et al. (2013), Streiner et al. (2015), and Wilson (2023)

Note: [R] indicates sub-steps that help to improve the reliability of the scale; [V] indicates sub-steps that help to improve the validity of the scale; while [R & V] indicates that the sub-steps help to improve both. Note that reliability is always a precondition for measurement validity.

## Preliminary phase

### Step 1: Need to measure a (clearly) defined construct

The initiation of scale development must be rooted in an actual clinical or research need to measure a certain clinical phenomenon.

The development of a new scale formally begins with the description of a construct (Clark and Watson, 2019). The construct is not something real in itself, it is an attempt to indirectly measure real phenomena (Edwards and Bagozzi, 2000). In psychometrics, constructs are sometimes termed “latent variables” since they are not directly observable but are inferred from measured reactions or behaviors, known as “observed variables.” For instance, in a psychological assessment, item responses (the observed variables) provide some indication about the underlying personality traits or psychopathology symptoms (the latent variables). In this sense, the concept of personality is not a real entity but is applied to a real clinical phenomenon. Since psychological and psychiatric characteristics (e.g., symptoms of psychopathology or personality traits) are latent constructs, their measurement relies upon the ability to make inferences based on responses to items about directly perceived inner experiences (self-report) or observed behaviors (both self-and clinician-report) to the characteristic of interest.

It is important to consider whether a certain construct is designed for universal applicability across cultures or is confined to a specific context. This decision influences whether an etic (universal) or emic (context-specific) approach is adopted for item development (Heggestad et al., 2019). The etic approach assumes the construct exists similarly across cultures and requires items to be generalizable, whereas the emic approach tailors items to specific cultural, social, or linguistic contexts (Vijver, 2010). In scale adaptation, the construct behind the measurement scale have to be validated prior to scale validation (Ambuehl and Inauen, 2022).

The definition of the initial construct domain should be theoretically grounded and clear enough to guide the early stage of scale development and to prevent accidental drift into unintended domains (DeVellis and Thorpe, 2022; Dimitrov, 2012; Irwing and Hughes, 2018; McCoach et al., 2013; Raykov and Marcoulides, 2011; Zickar, 2020). In cross-cultural contexts, this clarity helps ensure the construct remains relevant across different target groups. However, the definition of the initial construct is often somewhat broad and typically needs to be refined several times during the first steps of scale development (Irwing and Hughes, 2018).

A comprehensive review of the existing literature is fundamental (Boateng et al., 2018; Carpenter, 2018; Clark and Watson, 2019; Irwing and Hughes, 2018; McCoach et al., 2013) to obtain an in-depth understanding of the construct and its position within the existing theoretical framework, and to identify the gaps the new scale can fill (Haynes et al., 1995; McCoach et al., 2013). Higher levels of detail increase the validity of scale construction and mitigate issues associated with irrelevance or underrepresentation of content (Borsboom, 2009; Clark and Watson, 2019).

An important consideration during the conceptualization of a psychological construct is the dimensional nature (e.g., trait or state) and its level of measurement (e.g., nominal, ordinal, interval, or ratio). These aspects influence the format of the scale items, the design of the rating scale, and the choice of statistical techniques for scale validation (Embretson and Reise, 2013; Stevens, 1946).

Once the construct is defined, it is fundamental to formulate a theoretically or empirically grounded hypothesized model that indicates the expected factor structure and guides the factor analysis during scale validation (Brown, 2015; Haynes et al., 1995). Depending on the research goals, the scale can cover various aspects of a construct or focus on one specific aspect. For complex constructs with multiple dimensions, a multidimensional model may be hypothesized, resulting in a multifactorial scale. This is the case, for example, of the DSM-5 maladaptive personality trait model measured by the Personality Inventory for DSM-5 (Krueger et al., 2012), which assesses 25 personality trait facets including anhedonia, depressivity, and irresponsibility.

The process concludes with an explicit operational definition of the construct. The operational definition ensures distinctiveness, measurability, and psychological relevance of the construct (Netemeyer et al., 2003; Strauss and Smith, 2009) and provides a clear meaning for the construct and its dimensions (if any) and directs the development of items for the scale (Borsboom, 2009; Byrne, 2016), and the type of statistical analysis when the model’s structure will be validated.

It is important to clarify the distinction between scales and indexes. A scale typically measures a latent construct in which multiple items are aggregated to reflect an underlying, unobservable trait (e.g., personality, depression) (Streiner, 2003). In this case, the items act as effect indicators, meaning that they are correlated and represent manifestations of the same underlying concept. In contrast, an index combines observable indicators, or causal indicators, that directly define the components of a concept. Unlike scales, indexes do not assume a latent factor, and items may not be correlated, since each contributes independently to the overall construct (Streiner, 2003). This distinction is crucial because it influences how items are selected, how relationships among them are interpreted, and which statistical methods are appropriate for validating the tool. For example, scales rely on internal consistency measures such as Cronbach’s alpha or MacDonald’s omega, while indexes do not require such consistency (and often the coefficients would be low if an internal consistency measure were applied to an index).

### Step 2: Check for existing ones

Given that scale development is both time-consuming and costly, using an existing appropriate instrument is typically more practical. It is therefore important to verify that current scales do not already serve the intended purpose effectively. If existing scales do not align with the cultural or contextual needs of the target population, the adaptation of an instrument may be more suitable than creating a new one (Streiner et al., 2015). Introducing a new measurement tool always requires a strong justification, particularly in the presence of well-established instruments (Clark and Watson, 2019; DeVellis and Thorpe, 2022; Irwing and Hughes, 2018; McCoach et al., 2013; Streiner et al., 2015).

### Step 3: Overall planning

When the development of a new scale or adaptation of an existing one is necessary, the development process should be approached as a comprehensive research project that comprises multiple phases and steps, with a particular focus on data-driven decision-making (Roid, 2016). The preliminary planning step entails assembling the test development team, defining individual roles and duties, and elucidating the objective and structure of the test.

Recognizing that items and tasks appealing to developers may not engage examinees or ensure smooth administration for examiners, it is essential to conduct research with diverse participants (Roid, 2016). Extensive planning—usually lasting up to a year—may be required before finalizing the test design, with adjustments made as development progresses. Once these foundational decisions are established, a detailed timeline with specific deadlines for each step should be. This timeline should be flexible to revisions throughout the development process.

## Item development phase

### Step 4: Generate a large item pool

Items should be selected or devised to reflect the construct of interest (Clark and Watson, 2019; Dimitrov, 2012; McCoach et al., 2013). To avoid construct underrepresentation and construct-irrelevant variation, each item in the set should reflect a distinct facet of the construct and be sensitive to the true score of the latent variable (Messick, 1995; Nunnally and Bernstein, 1994). This can be the case of the so-called redundant items, where multiple instances of the same items are used to assess a specific part of the general construct. It can be argued that using only a single instance of an item may decrease the accuracy of detecting true responses (i.e., increasing the chances of false positives and false negatives); however, incorporating redundant items can enhance measurement reliability but may lead to respondent fatigue and increased dropout rates.

Item generation should incorporate both deductive and inductive methodologies (Hinkin, 2005). The first approach derives items from the construct’s theoretical definition and review of relevant literature and existing scales, while the second adopts an empirical bottom-up perspective that includes focus groups, key informant interviews, clinical observation, and others.

An initial phase of qualitative research to explore the experiences of the population of interest (typically, patients) may play a crucial role in the development of self-reported outcome measures (Food and Drug Administration, 2018, 2020). Eliciting and collecting participants’ lived experiences through, for example, individual interviews or focus groups (Ricci et al., 2019), can enrich the quality of scale items (McKenna, 2011) and improve content validity. More generally, target population engagement helps to understand the nuances and contextual factors that might influence the construct, thus ensuring a more holistic and representative item set.

Coupling modern technologies, such as machine learning and neural networks, to these traditional methodologies can widen the range of items and optimize existing ones, discovering potentially valid items that might have been overlooked by human experts (Götz et al., 2023; Netemeyer et al., 2003).

Collaborative projects, such as the International Personality Item Pool (IPIP) (https://ipip.ori.org) or the Patient-Reported Outcomes Measurement Information System (PROMIS) (https://www.promishealth.org), can be used as additional sources of freely accessible items; many of those are part of well-validated instruments (Streiner et al., 2015).

The language of items should be easily understood by the intended audience (Weiner, 2013), avoiding overly complex jargon (Gadermann et al., 2012) and ambiguity (Haladyna and Rodriguez, 2013; Krosnick, 2018), which could engender uncertainty among respondents. In particular, concise language – i.e., the “use as few words as possible in each item stem and options” (Haladyna and Rodriguez, 2013, p. 158) – is preferred over lengthy items because it reduces complexity and improves clarity and validity while preventing unnecessary repetition (Holden et al., 1985; Simms, 2008; Streiner et al., 2015). It also lowers reading level and reduces cognitive burden. Concurrently, focusing on the dimension of interest rather than the grammatical structure or specific wording of items, can enhance the understanding of the construct without watering down its essence (DeVellis and Thorpe, 2022).

Another essential aspect to consider is the word orientation of the items. It is common to employ both negatively worded items (which indicate low levels or the absence of the construct of interest) and positively worded items (which signal the presence of the construct). However, word reversals can potentially confuse respondents and thus lead to poor item performance (Chyung et al., 2018; Dalal and Carter, 2015). Therefore, it is recommended to ensure that the direction of the items corresponds to the majority and resonates with the understanding of the respondent.

An abundant initial pool of items, triple or quadruple the size of the final scale, allows a more judicious selection for the final scale and prevents poor internal consistency reliability (DeVellis and Thorpe, 2022; Streiner et al., 2015). It is crucial to align operational definitions back to their respective dimensions, guaranteeing a thorough content representation for each (McCoach et al., 2013). Items should provide full coverage of the content domain, including varying degrees of intensity. To accurately capture the core of each dimension, each subscale should comprise a minimum of three items (Clark and Watson, 2019; Costello and Osborne, 2005; Osborne, 2014), although four is the minimum to test that they are actually unidimensional, and it is better to start with an even larger initial set of candidate items to be able to pick the best from among them. Ideally, the number of items per dimension or subdimension ideally should correspond roughly to the significance of that idea within the broader dimension or construct’s definition.

### Step 5: Determine item structure and scaling of responses

The measurement format should be determined based on the studied construct and the scale objectives (DeVellis and Thorpe, 2022; Gadermann et al., 2012; Streiner et al., 2015). Choices such as the Likert scale, visual analog scale, or semantic differential scale significantly impact the reliability, validity, and perceived respondent load of the measurement (Preston and Colman, 2000).

#### Number of response categories

The number of response categories and scale length plays a pivotal role in data quality (Cicchetti et al., 1985; Preston and Colman, 2000). The selection between dichotomous (e.g., Yes/No), polytomous (multiple categories), or continuous response format can have profound implications on the psychometric properties of the scale. Generally, polytomous formats allow greater variability and a higher degree of precision in measurement and are more amenable to various statistical analyses compared to dichotomous formats (Bohrnstedt, 2010). A ‘thermometer’ scale, for instance, enables the measurement of states or traits from absolute absence to intense levels (Bollen and Lennox, 1991). Although it is vital to offer sufficient variability, respondents can be overwhelmed by excessive choices, potentially compromising data quality (Krosnick, 1991, 2018). An important decision involves choosing between an odd (which allows for a neutral response) or even (which mandates a choice) number of response options (Preston and Colman, 2000). The decision whether to label only the endpoints or each response option depends on the complexity of the construct and the literacy level of the respondents. Fully labeled scales are known to curtail ambiguity and bolster reliability (Bendig, 1954). Moreover, the construct’s assumed underlying structure should guide the choice of response categories. For constructs expected to follow a continuous distribution, it is recommended to use a greater number of response options to capture subtle variations (Preston and Colman, 2000). Conversely, if bimodal distributions are anticipated, using fewer response options might be more suitable to avoid artificial peaks in responses (Bohrnstedt, 2010). Additionally, the choice of response categories should reflect the potential for the construct to have a continuous versus a categorical underlying structure (Preston and Colman, 2000). Formats with more response options also may increase cognitive load, making two or three option formats sometimes preferable when respondents will be children, or responding in a second language. The number of response categories is one of the most important things to consider when defining a scale, since it can determine the following data analyses. This is the case of the Likert scale (see following subsection): it is well-known that such scaling is considered ordinal (at least until six categories). This means that, when validating the structure of the overall scale, a specific estimator for ordinal variable should be selected (e.g., the diagonally weighted least squares estimator) (Li, 2016).

#### Reversed items

Negated items are not the exact opposite of directly worded items. Mixing stems and response options is generally not advised, as it tends to be overly confusing for many respondents, especially those less motivated to respond (Weijters et al., 2013), and thus reduces the reliability and validity of scores (Barnette, 2000). Although the employment of reversed items can serve as an effective strategy to control for acquiescence bias, these should be used sparingly to avoid unnecessary confusion (Barnette, 2000). The risk of confusion is higher when using simple negations, such as including the modifier “not” in a stem (Swain et al., 2008). Furthermore, it is important to acknowledge that evidence indicates that negatively worded items demonstrate significantly lower discrimination (Sliter and Zickar, 2014) and that they can sometimes lead to a false two-factor structure when measuring what is intended to be a unidimensional construct (Brown, 2015; Schriesheim and Eisenbach, 1995). This is particularly relevant in factor analyses, where negatively phrased items may load onto a separate factor simply due to their wording, creating the illusion of multidimensionality when only one construct is being measured (Netemeyer et al., 2003).

#### Phrasing of items

Another crucial aspect in scale development is whether items are phrased as statements or questions. Statements generally prompt respondents to rate their agreement or disagreement, making them useful for measuring attitudes and beliefs. Questions, on the other hand, direct respondents to provide information or clarify behaviors, which makes them more suitable for factual or behavioral assessments (Streiner et al., 2015). The choice between these formats should align with the construct being measured and the type of responses expected from the target population.

#### Likert scaling

Likert-type scales should cover the measurement continuum with ordinal, nonoverlapping points (Krosnick and Presser, 2009). Since two-to three-point scales have lower reliability compared to five-to seven-point Likert-type scales, it is recommended to use five-point scales for unipolar items and seven-point scales for bipolar items (Krosnick and Presser, 2009; Rhemtulla et al., 2012). However, the Likert scale requires careful construction because extremely worded items may provoke extreme responses, obstructing differentiation among respondents. Balancing the forcefulness of item wording enhances the scale’s reliability (Schuman and Presser, 1996). Similarly, the use of even or odd number of categories can affect the respondents: it is sometimes preferable to use odd categories if it is necessary to fix a middle point (i.e., “Neither agree nor disagree”).

#### Frequency/intensity

The decision to measure the frequency and/or intensity of a construct is important in psychological and psychiatric assessment. These dimensions may reveal different patterns and relate to a construct’s dimensions in unique ways. For example, differing patterns of association have been found between personality traits measured by the NEO Personality Inventory: Revised (Costa and McCrae, 2008) and the frequency and intensity dimensions (Garcia and Erlandsson, 2011).

In defining and assessing symptom severity for most mental health disorders, both symptom frequency and intensity are typically considered (American Psychiatric Association, 2022; World Health Organization, 2022). Surprisingly, most self-report scales for psychiatric disorders focus solely on either intensity or frequency. This approach may not always be optimal. For example, to effectively assess DSM-5-based generalized anxiety disorder using the Generalized Anxiety Symptom Severity Inventory, it is necessary to incorporate both dimensions (Mordeno et al., 2021). On the contrary, for the evaluation of posttraumatic stress disorder with the modified PTSD symptoms scale (Falsetti et al., 1993), measuring either the frequency or intensity of symptoms is adequate (Elhai et al., 2006).

Choosing between these dimensions—or combining them—should be informed by the nature of the specific construct being measured. In cases of uncertainty, the safest approach is to collect data on both dimensions and defer the final decision until after the statistical analyses have been conducted.

#### Guttman and Thurstone scaling

Specialized methodologies for scale construction, such as Thurstone and Guttman scaling, use items that distinctly represent different attribute levels or increasing attribute levels, respectively (Dimitrov, 2012; McCoach et al., 2013). However, the idea of equally potent causal links between the latent variable and all items does not apply universally (Nunnally and Bernstein, 1994). For ordered items, item response theory-based models could provide a relevant, albeit complex, alternative (Baker and Kim, 2017).

#### Semantic differential scales and binary options

Semantic differential scales employ adjective pairs representing opposite ends of a continuum (Osgood and Tannenbaum, 1955). In contrast, binary options offer a simple choice for each item. Although format like usually yes/no or checked/unchecked simplifies responses, it restricts variability and covariation and often requires more items to achieve comparable scale variance.

#### Item time frame

The item time frame is an integral aspect of scale formatting and should be carefully considered. Scales can assess transient stable traits (e.g., trait anxiety) that remain consistent over time or transient phenomena (e.g., state anxiety) that fluctuate over short periods (McCoach et al., 2013). The choice of time frame (e.g., “over the past six months” or “in general” vs. “right now” or “today”) should be guided by the target construct and the intended purpose of the scale (DeVellis and Thorpe, 2022). For constructs that encompass both state and trait elements (e.g., mood disorders), multiple time frames may be considered to capture both fluctuations and long-term patterns. Clear examples of suitable time frames for different constructs ensure that the selected frame aligns with the theoretical basis of the measure and supports valid interpretation of the results.

#### General guidelines

Clarity in language and visual presentation is essential to prevent varied interpretations (Schwarz, 1999), especially for dichotomous response categories like true/false. The ability of respondents to distinguish between response categories is influenced by the attribute being measured, the specific phrasing, and the layout of the response options. Therefore, maintaining consistent polarity—such as using unipolar or bipolar scales consistently and preferring positive responses over negative ones—throughout the scale is vital to minimize respondent confusion and potential response bias (Weijters et al., 2010).

### Step 6: Design instructions for responding

Instructions are an essential, yet frequently underestimated, component of any inventory. They should be crafted with care to clearly articulate the response process, define the meaning of various points on the response continuum, and clarify the time frame the scale items are intended to investigate. Such clarity prevents confusion among respondents.

Instructions should be specific to the type of measurement scale being used. In Likert-type scales, instructions should clarify whether respondents are asked to indicate their degree of agreement or the frequency of behavior or experience. In more complex scaling methods, such as Thurstone or Guttman scaling, instructions must ensure that respondents comprehend the incremental nature of items or the criteria for choosing specific responses (McCoach et al., 2013).

Complex response formats, like visual analog scales or semantic differential scales, as well as assessments involving children, adults with limited literacy skills, or impaired cognitive functioning, should include practice items in the instructions (DeVellis and Thorpe, 2022; McCoach et al., 2013; Streiner et al., 2015).

Progression rules are another important aspect of well-designed instructions, especially for scales that involve multiple sections or levels of difficulty. These rules outline how respondents should proceed from one section to the next, ensuring that all items are addressed and that the order of items does not inadvertently affect responses (DeVellis and Thorpe, 2022). Progression rules are particularly relevant when using branching items, where subsequent questions depend on earlier responses (Irwing et al., 2018). Instructions must clearly outline how respondents should skip or move through sections based on their answers, reducing the risk of missing data or misinterpretation.

Finally, instructions should emphasize the time frame for responding. This is especially important when assessing transient psychological states, as ambiguity about the time frame could skew results. Clear definitions of the time frame (e.g., “in the past week” or “in the past month”) ensure that respondents focus on the relevant period when answering items.

### Step 7: Conduct an expert review

A thorough evaluation of the response instructions and the initial list of items by subject matter experts is vital to ensure clarity, relevance, and content validity. The expert review process is flexible and allows variations in the number of experts involved (typically three to ten), their selection process, and the approach to resolving any disagreements that might arise (Streiner et al., 2015). The panel of experts typically includes methodologists and content experts, both researchers and clinicians. The gathering of expert opinions can range from casual feedback on a draft version to quantitative judgmental rating tasks (DeVellis and Thorpe, 2022; McCoach et al., 2013; Ruel et al., 2016) or formalized meetings with prominent field leaders following established voting protocols (Boateng et al., 2018; Willis, 2005).

The strength of this approach is that if experts are chosen judiciously, they will likely encapsulate the most current insights in the field, offering scale developers access to a wealth of collective wisdom and experience (Clark and Watson, 2019). It is crucial to foster a broad spectrum of opinions among the expert panel to avoid the scale being dominated by a singular perspective (DeVellis and Thorpe, 2022), which could result in significant gaps in the final product. Even recommendations made by a single expert should be considered for the initial instrument draft (Gadermann et al., 2012). It is important to underline that despite the domain expertise of the panel members, some of them might not fully grasp scale development principles, which can occasionally lead to suboptimal suggestions (Willis, 2005).

### Step 8: Revise items and instructions

Revise instructions and items based on expert feedback. Occasionally, steps 7 and 9 may overlap, such as with scales measuring clinicians’ affective responses to patients like the Clinician Affective Response (CARE) Scale (Stefana et al., 2024a). In such cases, skip to step 10.

### Step 9: Conduct an evaluation by target population representatives

Conducting cognitive interviews (Beatty and Willis, 2007; Peterson et al., 2017) helps to identify and resolve potential ambiguities in assessment items as understood by representatives of the target population, typically involving five to fifteen participants (Peterson et al., 2017). This technique also allows for the assessment of validity evidence based on the respondents’ thought processes while formulating their responses (DeVellis and Thorpe, 2022; Streiner et al., 2015). In cross-cultural contexts, it is particularly important to ensure that items are understood as intended by different cultural groups. This involves addressing not only language translation but also conceptual and cultural relevance, which can vary significantly between populations (Heggestad et al., 2019).

Among the techniques that enhance the effectiveness of cognitive interviews for health scale development, the most useful in scale development (Collins, 2003; Foddy, 1993; Streiner et al., 2015) are: (i) paraphrasing/rephrasing (respondents restate the question in their own language), (ii) double interviewing (respondents complete the scale, and then are interviewed about the reasoning behind particular responses), (iii) thinking aloud interviews (respondents verbalize their thoughts during the response process), and (iv) probing (respondents are asked targeted follow-up questions that may address the difficulty in answering, the certainty, or their emotional reactions). The technique chosen is largely dictated by the nature of the item. Rephrasing or targeted probing is preferred for comprehension concerns, while think-aloud or double interview methods are optimal when recollection is essential (Streiner et al., 2015). These techniques, although extending the time required for completion, can be managed by asking each participant to respond to a subset of questions.

Preliminary testing of item sequencing is recommended to identify any sequencing-related issues, allowing for necessary adjustments before finalization (McCoach et al., 2013; Netemeyer et al., 2003). Additionally, especially in the case of translating or locally adapting a scale, recommended differential item functioning (DIF) procedures (Rouquette et al., 2019) should be employed to verify that the instrument functions equivalently across different target groups and languages (Krogsgaard et al., 2021). This helps reduce bias and ensures that items are not culturally or contextually misinterpreted.

## Scale construction phase

### Step 10: Create the final version of the survey

Revise the scale instructions and items based on feedback from representatives of the target population.

The sequencing of items is crucial for optimizing participant engagement and ensuring the reliability and validity of the results. Although research on item sequencing is somewhat limited and produces inconsistent findings (Lam et al., 2002; Şahin, 2021; Schell and Oswald, 2013), careful consideration of item order can enhance the psychometric properties of a scale. For scales that measure multiple dimensions or include items of varying valence, careful attention to item distribution is essential. For instance, in bidimensional or multidimensional scales, it is important to avoid clustering items from the same dimension together to reduce bias (DeVellis and Thorpe, 2022).

It is important to include a brief set of sociodemographic and clinical questions (such as diagnosis, duration of illness, and current treatments) to characterize the sample and provide initial validity evidence through their associations with the scale score (s). To avoid overwhelming participants, limit these questions to one side of a page. Refrain from using open-ended questions or responses, which are often skipped by respondents. Often responses are more complete if the demographics questions are placed at the end of the survey (Dillman et al., 2014).

Consider incorporating items that (i) detect possible biases, like social desirability, which could affect responses, and (ii) assess the relationship with related constructs, possibly eliminating the need for a separate validation procedure later. If you are using a social desirability scale, consider removing any item from your main inventory that shows a significant correlation with its score, unless there is compelling theoretical justification to retain it.

Format the survey to be professional in appearance, visually appealing, and user-friendly for readability (McCoach et al., 2013). If a questionnaire is easy on the eyes and easy to read, participants are more likely to participate and complete it (Dillman et al., 2014).

### Step 11: Administer to an appropriately large and representative sample

#### Sample size calculation

Several factors influence the determination of the required sample size, including item numbers, dimensions, variation between variables, level of overdetermination of the factors (i.e., the degree to which each factor is represented by a distinct set of items), and complexity of the model (MacCallum et al., 1999, 2001). Larger sample sizes or higher respondent-to-item ratios tend to produce lower measurement errors, more stable factor loadings, replicable factors, and results that are generalizable to the true population structure (MacCallum et al., 1999; Osborne, 2014). Inadequate sample size increases the likelihood of nonrepresentativeness, which can skew the resulting scale either quantitatively (narrower range of attributes) or qualitatively (different relationships among items or constructs) (Nunnally and Bernstein, 1994).

Regarding exploratory factor analysis (EFA), literature suggests rules of thumb consisting of minimum *N*s in absolute numbers like 100–250 (Cattell, 2012; Gorsuch, 2014) or 300 (Clark and Watson, 2019; Guadagnoli and Velicer, 1988; Tabachnick et al., 2019). Sample sizes has been graded as follows: 50 = ‘very poor’, 100 = ‘poor’, 200 = ‘fair’, 300 = ‘good’, 500 = ‘very good’, and 1,000 or more = ‘excellent’ (Comrey and Lee, 2013). However, these general thresholds do not consider the characteristics of the items and scales, even though these characteristics are more relevant than the absolute sample size (Osborne, 2014; Pett et al., 2003; Worthington and Whittaker, 2006).

The required sample size can be affected by factor loadings and communalities (i.e., the extent to which each individual variable contributes to the overall variance explained by a factor). If the factor loadings and communalities are low, it may be necessary to increase the sample size (Mundfrom et al., 2005). Communalities are generally considered high if above 0.80, though 0.40–0.70 is more frequent in social sciences (Costello and Osborne, 2005). Therefore, if all communalities are greater than 0.50 (or with at least 4:1 items per factor) and factor loadings are greater than. 40, samples smaller than 150 can be defended (Worthington and Whittaker, 2006). However, if communalities drop below 0.5, a larger sample size (≥ 300) becomes necessary to ensure statistical reliability. As the complexity of the model increases with more factors, the required sample size also increases (Bandalos and Finney, 2018).

Another category of rules of thumb is that of ratios. A minimum ratio of participants to items between 5:1 and 10:1 is commonly followed (Gorsuch, 2014), but others recommended 20 cases per variable for robust, generalizable results (Osborne, 2014). However, robust item loadings, consistent communalities, and the item-to-factor ratio are important to ensure the reliability, stability, and replicability of the factor solution (Osborne, 2014; Wang et al., 2013).

Regarding confirmatory factor analysis (CFA), a common rule of thumb for CFA recommends a ratio of cases to free parameters between 10:1 and 20:1 (Jackson, 2003; Whittaker and Schumacker, 2022). However, the process of determining the sample size for CFA should be multifaceted and dependent on numerous elements including, but not limited to, the temporal nature of the study design (longitudinal *vs* cross-sectional), interrelationships among indicators, the dependability of these indicators, scaling of data (continuous *vs* categorical), the estimator in use (e.g., ML, robust ML), missing data patterns, and the model’s intricacy (Brown, 2015; Kyriazos, 2018). Additionally, sample size depends on indicator reliability, with more reliable scales requiring smaller sample sizes to achieve adequate statistical power (Tabachnick et al., 2019).

Minimal sample sizes, informed by Monte Carlo simulation studies, aim to mitigate risks of nonconvergence and bias in estimations or standard errors. Despite CFA’s reputation as a large-sample methodology (Kline, 2023) smaller samples may suffice when dealing with robust parameter estimates and high-reliability variables (Tabachnick et al., 2019).

Power analysis must also factor in the sample size’s adequacy for achieving desired power in significance tests, model fit, and likelihood ratio tests pertinent to specific research contexts (Myors and Murphy, 2023; Wang and Rhemtulla, 2021). The influence of varying sample sizes on chi-square statistics, RMSEA, and other fit indices requires consideration as well (Hoyle, 2023; Hu and Bentler, 1999). It is imperative to maintain sufficient power for individual parameter tests, such as factor loadings, to ensure reliable and valid psychometric properties (Kyriazos, 2018).

It is therefore crucial to recognize that there is no single item-ratio that fits all scale development scenarios. The complexity and unicity of a given scale largely dictate the optimal sample size or the respondent-to-item ratio. However, it is widely accepted that larger sample sizes or higher respondent-to-item ratios are generally preferable. These conditions lead to lower measurement errors, more stable factor loadings, replicable factors, and results that are generalizable to the true population structure (MacCallum et al., 1999; Osborne, 2014). On the contrary, smaller sample sizes or lower ratios could result in more unstable loadings and factors, random, non-replicable factors, and results that may not be generalizable (MacCallum et al., 1999; Osborne, 2014).

Determining the appropriate sample size for exploratory graph analysis (EGA) depends on several factors, including the number of variables, the strength of inter-variable relationships, and the complexity of the network structure. Larger sample sizes generally lead to more accurate detection of latent dimensions and more stable estimation of partial correlations (Christensen et al., 2020; Golino and Epskamp, 2017). Although there is no fixed rule for determining sample size, simulation studies suggest that networks with more variables or weaker relationships between them require larger samples. For complex networks, a sample size of 500 or more observations is typically recommended to ensure stable and accurate results, while simpler networks may yield reliable results with 250 observations (Golino et al., 2020). However, when the latent structure is more intricate or relationships between variables are weak, larger sample sizes are necessary to avoid misidentifying community structures (Christensen et al., 2020). Recent studies emphasize the importance of algorithm selection in community detection, which can influence network stability. For instance, the Walktrap algorithm, commonly used in EGA, performs well in detecting communities but may struggle with unidimensional structures. This limitation led to the development of a unidimensionality adjustment to improve accuracy (Christensen et al., 2023; Golino et al., 2020). This adjustment, along with bootstrapping, allows for better evaluation of dimensional stability by identifying inconsistencies in community detection across samples (Christensen and Golino, 2021a). Stability assessments, such as bootstrapping, are recommended to ensure the identified network structure is consistent across varying sample sizes. The bootstrapping method, known as bootstrap exploratory graph analysis (bootEGA), evaluates the stability of dimensions and items across bootstrap replicates, providing insights into whether the network dimensions generalize to other samples (Christensen and Golino, 2021a). These assessments typically suggest that sample sizes of 500 or more are ideal for robust community detection and network estimation (Golino et al., 2020). In summary, while EGA generally requires larger sample sizes than traditional factor analysis methods due to its reliance on partial correlations, the literature suggests that 500 observations is often a reasonable target for reliable network estimation, particularly for complex networks. Additionally, the inclusion of bootstrapping techniques further enhances the robustness of EGA results, ensuring stability in community detection (Christensen and Golino, 2021a).

In general, to demonstrate the scale’s generalizability, replicating a factor-analytic solution on a separate sample remains the best approach (DeVellis and Thorpe, 2022). Having the second sample be from a different geographic location or use a distinct recruiting strategy further enhances generalizability of findings (König et al., 2007; Youngstrom et al., 2018).

#### Administration

At a minimum, scale development requires data collected from a single sample. However, to thoroughly evaluate the scale’s dimensionality and internal consistency, data should also be collected from an independent sample. Alternatively, data can be collected from the same sample at different time points: baseline data can be used for initial scale development and to perform a first CFA, while follow-up data can be used to perform a second CFA and evaluate test–retest reliability. This longitudinal approach can increase the risk of common error variance because using the same participants and measures over time may introduce consistent response patterns and method biases.

Regarding the modes of survey administration, data can be gathered through multiple ways such as self-administrated paper-and-pencil, face-to-face or telephone interviews, and lab-based or online-based devices.

The use of technology-based survey methodologies is recommended whenever possible (DeVellis and Thorpe, 2022). They can significantly reduce data entry errors, improve response rates, provide immediate feedback, and facilitate the collection of data from larger samples at lower costs (Anwyl-Irvine et al., 2021; Gosling and Mason, 2015; Regmi et al., 2017). Multiple web-based platforms, such as Research Electronic Data Capture (REDCap), are available to create digital forms. These web-based platforms comply with data general protection regulations, ensuring the security and privacy of participant data (Van Bulck et al., 2022). Furthermore, certain softwares for data collection, such as the Questionnaire Development System™, allow to capture audio data, improving accessibility for participants with impaired vision or low literacy levels.

While the paper-and-pencil method is more laborious and susceptible to human error, it can be advantageous in specific situations (Dillman et al., 2014). For instance, it is often more effective with older populations, including healthcare professionals, who are more likely to respond to paper surveys than to digital ones (Ernst et al., 2018; Hardigan et al., 2016).

### Step 12: Extract the factors

As a first step, the suitability of the data for factor analysis must be evaluated using Bartlett’s sphericity test (*p* ≤ 0.05) and Kaiser-Meyer-Olkin measure of sampling adequacy (KMO ≥ 0.60) (Shrestha, 2021).

A combination of theoretical reasoned reflection, parallel analysis (Horn, 1965) with replications of the simulated comparison data, and visual scree test (Cattell, 1966; Horn and Engstrom, 1979) should be used to determine the exact number of factors to retain (Carpenter, 2018; DeVellis and Thorpe, 2022). Further methods that can be implemented are the minimum average partial (Velicer, 1976; Velicer et al., 2000), the Hull method (Lorenzo-Seva et al., 2011), and other simulated comparison data methods (Goretzko and Ruscio, 2023; Ruscio and Roche, 2012). It is common for researchers to use multiple methods to arrive at a final decision, as many software packages provide several indices for this purpose. Importantly, when employing various alternative procedures, it is essential to avoid selective reporting. All measures should be reported, and the choice of analysis must be justified using both the data and theoretical rationale to avoid bias in interpretation (Zygmont, 2023; Zygmont and Smith, 2014).

#### Rotation method

Rotation methods in factor analysis can be broadly classified as orthogonal (producing uncorrelated factors) and oblique (yielding correlated factors). The choice between the two should be based on whether the dimensions of the study construct are theorized to correlate. In the absence of such a theory, oblique rotations generally offer more accurate data representations, as psychological/psychiatric constructs are often interrelated. However, if the factors are not correlated, an oblique rotation will produce an orthogonal solution, which presents no loss (Gorsuch, 2014; Thompson, 2004).

Varimax (orthogonal) rotation is the most commonly used rotation method used in statistical analysis (Akhtar-Danesh, 2017), but may not be the optimal choice as it does not allow factor correlation, which is common in social and mental health sciences (DeVellis and Thorpe, 2022). It can also generate more cross-loadings and lessen the likelihood of identifying a general factor when present (Irwing et al., 2018). Oblimin and Promax (oblique) rotations offer better representations, particularly if factors correlate substantially (Irwing et al., 2018). Although both methods allow factor correlation, Promax starts with an orthogonal solution before transforming it into an oblique one, making it more robust.

#### Exploratory factor analysis

Exploratory factor analysis (EFA) is a hypothesis-generating technique that helps to determine the underlying factor structure of the inventory. By examining the relationships among the items, it provides valuable insight into which factors best account for the variation observed. If the results of the EFA do not align with the expected theoretical structure, it may be necessary to go back to the initial steps of the scale development process (McCoach et al., 2013).

When interpreting EFA results, both factor pattern coefficients and factor structure coefficients must be considered (Henson and Roberts, 2006; Thompson and Daniel, 1996). These coefficients indicate the contribution of a variable to a specific factor. The factor structure matrix reveals the correlations between all the observed variables and the extracted factors. With orthogonal rotations, these factors remain uncorrelated and both matrices match. In contrast, for oblique rotations where factors correlate, the structure matrix does not equal the pattern matrix, necessitating interpretation from both (Courville and Thompson, 2001; Henson and Roberts, 2006).

#### Exploratory graph analysis

As a complement or substitute for parallel analysis and EFA, exploratory graph analysis (EGA) offers a viable approach (Golino et al., 2020; Golino and Epskamp, 2017). EGA produces comparable accuracy or even outperforms other traditional factor analytic methods in correctly estimating the number of dimensions (referred to as “communities” in its nomenclature) for continuous data (Christensen et al., 2023; Cosemans et al., 2022; Golino et al., 2020). Furthermore, EGA can provide a more interactive and visually intuitive analysis of data dimensions. EGA uses cluster detection on estimated psychological networks to identify dimensions that are statistically equivalent to latent variables (Christensen and Golino, 2021b; Golino and Epskamp, 2017). EGA focuses on direct item relationships within dimensions, eschewing the need for latent variable assumptions. Items within a dimension are assumed to be more strongly associated with each other than with those of a different dimension, and covariation among items is not assumed to be caused by an unobserved latent variable. This implies that EGA concentrates on the direct relationships between items for dimension identification. This approach operates in a data-driven way, thus eliminating the need for factor rotation decisions, further simplifying the analytical process and making it particularly effective in identifying unique factors even when correlations among them are high (Heshmati et al., 2022). Furthermore, EGA automatically allocates items to a dimension, bypassing the need to interpret a factor-loading matrix. Lastly, EGA provides a color-coded network plot for a straightforward interpretation of factor-item relationships (Bringmann and Eronen, 2018).

EGA allows the quantification of item stability, dimension stability, and structural consistency, calculated on a scale ranging from 0 to 1. Item stability is the frequency of each item’s allocation to each of the detected dimensions and offers insights into potential sources of structural inconsistency. It ranges from 0 (completely unstable) to 1 (perfectly stable) with a cutoff of 0.65 (Christensen and Golino, 2021a). Dimension stability refers to the frequency of replication of the same number of dimensions and employs network loadings (calculated as the total sum of all edge weights for a node within each dimension). Thresholds of 0.15 for small, 0.25 for moderate, and 0.35 for large effect sizes have been suggested (Christensen and Golino, 2021b). The network loadings matrix is useful for pinpointing items that demonstrate cross-loading or multidimensionality (Christensen et al., 2020). Structural consistency offers an alternative to traditional internal consistency in latent models. It ranges from 0 (structural inconsistency) to 1 (identical item composition across all bootstrap samples), with values of 0.75 or higher regarded as acceptable (Golino et al., 2021). Thus, EGA’s comprehensive approach yields a nuanced and detailed understanding of the relationships between items and dimensions within a dataset.

### Step 13: Identify the best items

To identify functional items, classical (test-level) and modern (both item-level and test-level) test theories can be used together to balance the weaknesses of each other (Boateng et al., 2018; Streiner et al., 2015). The choice of which combination of specific techniques to utilize should be tailored to the scale’s particular characteristics. In any case, item reduction analysis should balance the potential improvement in psychometric performance against the cost of losing potentially meaningful information from the scale. Furthermore, it should also be based on the theoretical relevance of the item and its coherence within the conceptual framework of the scale (DeVellis and Thorpe, 2022; McCoach et al., 2013).

It is crucial to avoid redundancy and select diverse yet representative items that represent unique aspects of the latent factor, reflecting the complexity of the construct without sacrificing brevity (Carpenter, 2018). To accurately capture the core of each dimension, each subscale should comprise a minimum of three items (Clark and Watson, 2019; Costello and Osborne, 2005; Osborne, 2014). Two-item scales should generally be endorsed only when items have a high correlation (*r* > 0.70) (Worthington and Whittaker, 2006).

#### Outliers

Outliers can distort the results of factor analysis and other item-level analyses, leading to biased estimates and incorrect conclusions (Streiner et al., 2015). Therefore, outlier detection methods, such as standardized residuals, leverage values, or Mahalanobis distance, should be employed to identify extreme values (DeVellis and Thorpe, 2022). If outliers are detected, researchers should carefully assess whether to remove them or apply transformations to minimize their influence without compromising the integrity of the data (Kyriazos and Stalikas, 2018). Outliers should be kept unless there is clear evidence showing that they are genuinely anomalous and do not reflect any observations within the target population (Hair et al., 2022).

#### Multivariate normality

Assessing the assumption of multivariate normality is important because many statistical techniques, such as the maximum likelihood in confirmatory factor analysis, assume normally distributed multivariate data (Li, 2016; Nunnally and Bernstein, 1994). Violations of this assumption can affect the accuracy of parameter estimates, standard errors, and fit indices (Mulaik, 2010). To check for MVN, skewness, kurtosis, and multivariate outliers should be evaluated. In cases of significant non-normality, techniques such as bootstrapping, robust maximum likelihood estimation, or data transformation may be employed to handle deviations from multivariate normality (DeVellis and Thorpe, 2022; Enders and Baraldi, 2018). Ensuring that the data meet these assumptions, or using appropriate remedies when they do not, enhances the validity and reliability of the scale development process (Wilson, 2023).

#### Factor loadings and slope coefficients

Items with factor loadings or slope coefficients less than 0.30 are deemed insufficient because they contribute less than 10% variance to the latent construct measured, a threshold often used to ascertain minimal significant contribution (Pett et al., 2003; Raykov and Marcoulides, 2011; Russell, 2002). However, higher factor loadings have been suggested as more reliable: 0.32 (Carpenter, 2018; Worthington and Whittaker, 2006), 0.35 (Clark and Watson, 2019), 0.40 (Hair et al., 2022; Reinard, 2006), and 0.50 (Mertler and Vannatta, 2016), depending also on the scale’s focus (narrower vs. broader) (Clark and Watson, 2019).

Items presenting cross-loadings or not loading distinctly on individual factors can be problematic, as they might hint at multicollinearity, shared variance, or issues with construct validity. Therefore, their removal is often recommended. However, it is important to consider the nature of the construct. If the construct is a circumplex (e.g., as in models of emotions or colors), cross-loadings are expected because items may inherently span multiple factors across any rotation of a two-dimensional mapping. In such cases, cross-loadings are not necessarily indicative of poor psychometric properties but rather reflect the theoretical structure of the construct. Consistent with this, minor cross-loadings—where the difference between loadings is less than 0.10 and at least one loading is greater than 0.30—might not significantly detract from the clarity or validity of the factor structure (Hair et al., 2022; Tabachnick et al., 2019). Hence, retaining such items can enhance the richness and comprehensiveness of the data, particularly in the context of multidimensional constructs like circumplex models.

#### Correlations

Higher correlations among items contribute to stronger correlations between individual items and the true score of the latent variable, enhancing overall scale reliability (Crocker and Algina, 2008; DeVellis and Thorpe, 2022). Intercorrelation can be assessed through the correlation matrix (Pituch and Stevens, 2016). While the primary selection is based on correlation patterns, evaluating means and variances serves as a useful cross-check (Nunnally and Bernstein, 1994). Items with low variances are less able to have meaningful covariances with other items.

However, extremely high correlations among items require attention. While high intercorrelations can contribute to internal consistency, they do not necessarily ensure that the items measure a single underlying construct (Dimitrov, 2012). Very high correlations might indicate redundancy, potentially compromising the validity of the factor structure (DeVellis and Thorpe, 2022).

#### Considerations for evaluating inter-item correlations

Inter-item correlations (which include tetrachoric correlations for binary items and polychoric correlations for categorical variables) serve a dual purpose. They assess (a) how closely one item’s score is correlated to the scores of all other items within a scale and (b) how consistently items reflect the same content domain (Cohen et al., 2013; Raykov and Marcoulides, 2011). Items with *r* < 0.30 might not be optimal and might need to be considered for removal from the scale (Cohen et al., 2013). The average interitem correlation (AIIC) should fall within the range of 0.15–0.50, ensuring a balance between desirable commonality and the avoidance of redundancies between items (Briggs and Cheek, 1986). Lower thresholds may lead to too much heterogeneity, while exceeding the upper limit may imply item redundancy. However, for a broad higher order construct such as extraversion, an AIIC as low as 0.15–0.20 may be appropriate; however, for a scale that measures a narrower construct such as anger rumination, a much higher AIIC (e.g., 0.40–0.50) is required (Clark and Watson, 2019). AIIC is a more insightful and direct measure of scale homogeneity than Cronbach’s alpha. The inter-item correlation is more useful than alpha for short scales, as it is unrelated to scale length (Streiner et al., 2015).

Negative item correlations suggest opposing item sentiments within the same construct, necessitating reverse scoring. If negative correlations persist after this adjustment, it may signal lack of alignment with the scales construct, requiring removal (Clark and Watson, 2019).

#### Item-total correlations

Item-total correlations (biserial correlations for binary items and polyserial correlations for categorical variables) evaluate the correlation between each item and the cumulative scale score (Raykov and Marcoulides, 2011). These correlations generally should be corrected by excluding the item in question to avoid the risk of inflating the correlation coefficient. Items with very low adjusted item-total correlations (< 0.30) are not optimal and could indicate a need for potential removal from the scale. More generally, items with higher values are more desirable than items with low values (DeVellis and Thorpe, 2022). This index is particularly relevant when testing the internal consistency and the reliability of the new measure.

#### Item variance

Relatively high item variance signifies effective discrimination among respondents with different levels of the measured construct (DeVellis and Thorpe, 2022). This also assumes equal covariances across items with the true score (Raykov, 2001). Items with a variance close to 0 must be removed.

#### Item means

The item means should ideally hover near the midpoint of the range of possible scores (DeVellis and Thorpe, 2022). Extremes can suggest potential item bias or the failure to capture certain construct aspects. If item response theory is used, then these items can be evaluated to see if they are informative at low or high levels of the latent trait.

#### Missing data

Scale developers must inspect patterns of missing data, determine an acceptable level of missingness (e.g., 20% per item on any given subscale per participant), and decide whether to impute missing values or use available item analysis (Parent, 2013). For imputation, full information maximum likelihood (Enders and Bandalos, 2001) and item-level multiple imputation (Gottschall et al., 2012; Kenward and Carpenter, 2007) have demonstrated considerable utility. Arbitrary cutoffs should be avoided to prevent biased results (Schlomer et al., 2010).

Handling (sub)scale-level missingness, where participants exceed the item-level missingness tolerance threshold, requires different strategies. Listwise deletion is recommended when the loss of participants is minimal (e.g., less than 5%) and scale-level imputation offers only a marginal increase in sample size (Parent, 2013; Schafer and Graham, 2002). For all other situations, multiple imputation should be employed (Parent, 2013).

Researchers should clearly report the level of missing data, specifying the tolerance level and the percentage of missing data by subscale and per participant. Ensure transparency by detailing missingness patterns and checking for any abnormal spikes in missing rates (Schlomer et al., 2010).

For item-level missing data, consider using available item analysis instead of participant mean substitution or multiple imputation, especially when missing data levels are below 10%. Available item analysis can be effective if the analysis focuses on scale means rather than item-level responses (Parent, 2013).

#### Item response theory

Most of the previously discussed steps are based on classical test theory (CTT), which assumes that an observed score is the sum of a true score and random error. An alternative approach is item response theory (IRT) (Baker and Kim, 2017; Wilson, 2023), which differs from CTT by focusing on the interaction between an individual’s latent trait (e.g., depression severity) and item characteristics (e.g., difficulty and discrimination). Unlike CTT, which operates at the test level, IRT operates at both the item and person levels, providing a deeper understanding of how individuals respond to specific items. IRT examines the relationship between a latent trait, such as depression or a maladaptive personality trait, and the probability of certain responses to test items. For instance, individuals with a higher level of the trait (e.g., depression) are more likely to endorse an item reflecting severe depressive symptoms (Foster et al., 2017).

One of the key advantages of IRT over CTT is its extension beyond dichotomous response scales to include polytomous or multitiered response scales, such as Likert scales, visual analog scales, and adjectival scales—which use descriptors along a continuum, with or without numbers under the words, rather than solely labeling the endpoints (Streiner et al., 2015). This feature is valuable since many psychological and psychiatric scales offer a range of responses rather than a simple yes/no or true/false option. IRT accommodates polytomous responses, which are common in psychological assessments where responses reflect varying degrees of agreement or severity. The graded response model (GRM) is particularly suited for this type of data, making it a popular choice in psychological and organizational research (Foster et al., 2017; Samejima, 2010).

The mathematical models used in IRT differ based on the parameters they estimate. The simplest model, the Rasch model (or one-parameter logistic model), estimates only item difficulty. The two-parameter logistic model accounts for both item difficulty and discrimination, while the three-parameter logistic model incorporates a guessing parameter to acknowledge the chance of a correct response due to guessing (Baker and Kim, 2017). For scales with multiple response categories, polytomous models such as the partial credit model (PCM) and the graded response model (GRM) are commonly applied (Masters, 2010; Samejima, 2010). These models, extensions of the one-and two-parameter logistic models, assume varying distances between response options (GRM) or equal distances (PCM). The GRM is generally favored for its better reflection of reality (Samejima, 2010; Streiner et al., 2015). It treats each item as if it were a scale with multiple items, each with its own thresholds. All threshold response curves for a particular item are assumed to have the same slope or discriminating ability, meaning each item can have different discriminating abilities. This model reveals that larger thresholds may exist between certain response options, providing a more nuanced understanding of respondent behavior, which is critical in clinical and psychological assessments.

A crucial aspect of applying IRT models is determining the appropriate sample size, directly impacts the precision of parameter estimates and the reliability of the findings. General recommendations suggest a minimum sample size of 150–250 for stable parameter estimates in unidimensional IRT models (Zickar, 2020). However, more complex models or those with polytomous responses typically require larger samples (Bock and Gibbons, 2021). For instance, simulation studies suggest that models like the GRM may require sample sizes of at least 300 to achieve robust estimates (Dai et al., 2021; Foster et al., 2017; Schroeders and Gnambs, 2024). Advancements in estimation techniques, such as Markov Chain Monte Carlo (MCMC), have reduced the sample size requirements for IRT models, making them more feasible for use in psychological research (Foster et al., 2017). Despite these advancements, researchers are still advised to conduct simulation-based power analyses to determine optimal sample sizes based on their specific research conditions (Schroeders and Gnambs, 2024), including the length of the test, the number of response categories, and the complexity of the model being used.

#### Item discrimination index

Item discrimination (*α* parameters) measures how well an item differentiates between individuals with varying levels of the latent trait. A discrimination parameter value of 0.01–0.34 is ‘very low,’ 0.50–0.64 is ‘low,’ 0.65–1.34 is ‘moderate,’ 1.35–1.69 is ‘high,’ and > 1. 70 is ‘very high’ (Baker and Kim, 2017).

The item characteristic curve (ICC) or item response function (IRF) visually depicts the relationship between the latent trait and the probability of a certain item response. ICC typically takes the form of an S-shaped logistic function, demonstrating that as a person’s trait level increases, the likelihood of consistently supporting an item or achieving it increases. The steepness of this curve indicates the discrimination property of the item.

#### Item difficulty index

Item difficulty (*β* parameters) signifies the level of the latent trait in which an individual has a 50% chance of endorsing an item or performing it correctly, thus indicating how ‘difficult’ or ‘easy’ an item is (DeMars, 2010). Scale developers must determine the appropriate difficulty level for their needs: for instance, when developing general-purpose scales, one typically focuses on items with medium difficulty (de Ayala, 2022). For polytomous items, there is a curve for each shift between response options, which can be plotted as option characteristic curves.

## Scale evaluation phase

### Step 14: Test the factor structure

The collective nature of items does not inherently constitute a scale. The optimal statistical method to test the nature of the latent constructs that underly the variables of interest is confirmatory factor analysis (CFA) (Bandalos and Finney, 2018). However, to confirm that the scale works equivalently across different groups, such as cultures or demographic categories, measurement invariance testing must be conducted (Leitgöb et al., 2023; Maassen et al., 2023).

CFA is a hypothesis-testing approach based on structural equation modeling (Norman and Streiner, 2014). This method hinges on a strict independent clusters model, which presumes that cross loadings between items and nontarget factors are exactly zero (Morin et al., 2016a). The most common techniques and respective satisfactory thresholds for testing factor structure are the following: chi-square divided by degrees of freedom (χ^2^/*df*) ≤ 2 (Alavi et al., 2021) Comparative Fit Index (CFI ≥ 0.95), Tucker Lewis Index (TLI ≥ 0.95), Root Mean Square Error of Approximation (RMSEA ≤0.06), Standardized Root Mean Square Residual (SRMR ≤0.08) (Hu and Bentler, 1999; Kline, 2023). However, it is important to note that these thresholds are general guidelines (Hoyle, 2023). Therefore, they are not universally applicable across all models. Their sensitivity varies depending on factors like sample size, the number of items, and factor loadings (McNeish and Wolf, 2023a). The original cutoffs by Hu and Bentler (1999) were derived from models with omitted cross-loadings or covariances, which may not apply to simpler models, like one-factor models (McNeish and Wolf, 2023b). Therefore, instead of relying solely on fixed cutoffs, researchers are encouraged to develop model-specific cutoffs using simulation-based methods. The *dynamic fit index cutoffs* approach (Wolf and McNeish, 2023) facilitates this process by allowing for the computation of fit indices tailored to the specific characteristics of a model, providing more accurate and meaningful evaluations of fit. This approach is particularly useful in models where traditional misspecifications, such as omitted cross-loadings, do not apply. The developers have made a Shiny R application available with a point-and-click interface for users to be able to get dynamic fit indices customized for their data and model (https://dynamicfit.app/) See also the simulation-cum-ROC (Goretzko et al., 2022) and ezCutoffs (Schmalbach et al., 2019) approaches.

Confirmatory bifactor modeling, also known as nested factor modeling, serves as an effective tool to examine the factor structure of a scale (Reise et al., 2023). This approach is particularly useful when a proposed factor structure results in partially overlapping dimensions (Brown, 2015). The bifactor model posits that each item is associated with two separate dimensions, indicating that the items that construct the latent variable could be linked to multiple sources of true variance of scores (Morin et al., 2016a). The first dimension represents a pervasive general latent factor that influences all scale items, while the second dimension consists of group factors or subscales. For example, the Toronto Alexithymia Scale (Bagby et al., 1994) is composed of three subscales that assess externally oriented style of thinking and difficulties in identifying and describing feelings. Its total score does measure a single construct, while the nested factors describe specific facets of the alexithymia personality construct (Carnovale et al., 2021).

The bifactor model enables a thorough examination of potential inconsistencies that arise when unidimensional IRT models are applied to multidimensional data (Embretson and Reise, 2013; Reise et al., 2023). The determination of a construct’s unidimensionality or multidimensionality involves comparing factor loadings from the general factor with those from the group factors (Chen et al., 2012; Reise et al., 2023). The bifactor model also provides a method for evaluating both a general factor underlying the construct and multiple group factors that explain the remaining variance not covered by the general factor (Rodriguez et al., 2016). Furthermore, it helps to distinguish between construct-relevant multidimensionality and construct-irrelevant psychometric multidimensionality, which is crucial for accurate interpretation of scale scores (Reise et al., 2023; Rodriguez et al., 2016). The effectiveness of the model is evaluated on the basis of predefined thresholds (Morin et al., 2016a). Bifactor CFA should be employed when the theory supports the conceptualization of two layers of constructs (Alamer, 2022; Morin et al., 2020; Tóth-Király et al., 2018). “In psychiatric, epidemiological and biomedical research, (…) bifactor models provide a more flexible, realistic, and meaningful representation of the data whenever these dimensions are assumed to reflect a global underlying construct,” compared to first-order or higher-order EFA or CFA (Morin et al., 2016b, p. 285).

Another possible approach is exploratory structural equation modeling (ESEM). It is a technique that combines aspects of EFA and CFA, thereby enabling the verification of preestablished factor structures (Marsh et al., 2014). A key attribute of ESEM is its capacity to handle cross-loadings, which allows items to be associated with several factors. This approach effectively restricts non-primary associations between items and factors to near-zero, avoiding exaggerated parameter estimates or misrepresentations of model fit. ESEM can be applied using Geomin rotation or targeted rotation. Geomin rotation takes an explorative approach, fixing a specific number of latent factors and allowing the algorithm to identify primary loading items for each factor (Prokofieva et al., 2023). On the contrary, the targeted rotation focuses on hypothesis testing, accommodating cross-loadings in the hypothesized model framework. It evaluates the targeted items in light of their primary dimension and other pertinent dimensions. Incorporating both methods into ESEM increases the precision and integrity of factor structure analysis. ESEM, along with the wider bifactor-ESEM framework, facilitates a more accurate portrayal of the construct-relevant psychometric multidimensionality inherent in many measures (Hoyle, 2023). Traditional CFA methods, overlooking this multidimensionality, fail to accurately define the latent constructs of interest, leading to overestimated factor correlations as compensation for the unacknowledged conceptually related and hierarchically structured nature of the constructs (Asparouhov et al., 2015; Morin et al., 2016a).

The two methods can be combined: a bifactor structure can be specified with ESEM modeling the cross-loadings and minor loadings. It is important to note that bifactor ESEM becomes the preferred approach under two specific conditions: (i) when there is a global underlying construct that influences all indicators or items, and (ii) when the items correspond to more than one source of true score variance (Morin et al., 2016a). Both conditions represent sources of construct-relevant psychometric multidimensionality.

Measurement invariance is typically tested through multiple-group confirmatory factor analysis (MGCFA) (Putnick and Bornstein, 2016), which involves three primary stages with increasing levels of restriction: configural invariance, which tests whether the same factor structure holds across groups; metric invariance (also known as weak invariance), which examines whether factor loadings are equal across groups; and scalar invariance (also known as strong invariance), which tests for equality of item intercepts across groups (Xu and Tracey, 2017). It is often evaluated by changes in fit indices, with acceptable thresholds including a change in CFI and TLI of less than 0.01, RMSEA below 0.015, and SRMR below 0.03 (Chen, 2007). If these conditions are satisfied, the scale can be considered invariant, allowing for meaningful comparisons of latent constructs across different groups.

Additionally, because the traditional approach to multi-group CFA does not estimate the effect size of item bias, researchers can consider using an effect size measure for differences in CFAs’ means and covariance structures (*d*_MACS_) (Nye and Drasgow, 2011). This method complements significance testing and helps quantify the magnitude of measurement non-equivalence at the item level. Empirical benchmarks for interpreting effect sizes have been developed to serve as guidelines rather than rigid cut-offs: dMACS values ranging from 0.20 to 0.40 indicate small measurement non-equivalence, values between 0.40 and 0.70 indicate medium non-equivalence, and values of 0.70 or higher indicate large non-equivalence (Nye et al., 2019).

Given that achieving full invariance can be particularly challenging in clinical and cross-cultural research (Leitgöb et al., 2023; Stefana et al., 2025), researchers may also consider using the alignment method (Asparouhov and Muthén, 2014) as a more flexible alternative (Luong and Flake, 2023). This method allows for assessing metric and scalar invariance without enforcing strict equality constraints on factor loadings or intercepts across groups, offering a practical alternative when full scalar invariance cannot be achieved.

### Step 15: Test reliability, agreement, and measurement precision

The length of the scale serves as a fundamental determinant of the reliability of the scale (Revelle and Condon, 2019; Streiner et al., 2015). Longer scales tend to engender higher reliability coefficients, partly due to the increase in shared variance among items (de Vet et al., 2011), although alpha and some other coefficients also have item count in their formula.

#### Internal consistency reliability

As noted earlier, internal consistency is a type of reliability pertinent to scales, but not to indexes (such as life events scales) that do not have an underlying latent factor. Cronbach’s alpha (Cronbach, 1951) is the most widely used estimate of internal reliability in counseling research (Kalkbrenner, 2023). It measures the extent to which items within a scale consistently assess the same underlying construct, but its assumptions and limitations must be acknowledged. A key assumption of Cronbach’s alpha is tau-equivalence, which posits that all items on a scale have the same true score variance. However, in practice, this assumption is rarely met (Revelle and Condon, 2019; Sijtsma, 2009). Consequently, alpha may either overestimate or underestimate reliability, depending on the scale’s structure and whether its items meet tau-equivalence (Raykov, 1997). Additionally, Cronbach’s alpha is sensitive to violations of multivariate normality, which can further distort reliability estimates (Trizano-Hermosilla and Alvarado, 2016). Of great conceptual concern, alpha assumes that a single factor underlines the item set, making it conceptually inappropriate when the instrument might have multiple subscales, or the item set might reflect multiple factors (Revelle and Condon, 2019; Youngstrom et al., 2019). Selecting items to maximize alpha also will create narrower content coverage and poorer construct representation—things to be particularly mindful of when constructing short forms and brief scales (Streiner et al., 2015; Youngstrom et al., 2019).

Given these issues, McDonald’s omega (McDonald, 1999), derived from factor analysis, is often a more appropriate reliability estimate. Omega accounts for the general factor saturation of a scale and is less likely to inflate reliability for multidimensional scales (Revelle and Condon, 2019; Zinbarg et al., 2005). We recommend reporting both alpha and omega values (at least for total scores), as omega typically provides a more accurate reflection of true reliability, while alpha allows for comparisons with prior research. It is important to note that Cronbach’s alpha does not always overestimate reliability; under certain conditions, it may underestimate it. Therefore, relying solely on alpha could misrepresent a scale’s internal consistency (Sijtsma, 2009).

Reporting omega alongside alpha offers a more comprehensive reliability assessment and reduces the risks associated with the uncritical use of alpha. Alpha and omega values usually range between 0.00 and 1.0, with a threshold of 0.70 as adequate, 0.80 as good, and 0.90 as excellent (Youngstrom et al., 2017). Negative values could indicate issues such as negative item correlations that need to be addressed.

In some cases, Revelle’s beta (Revelle and Condon, 2019) or the greatest lower bound (GLB) (Bentler and Woodward, 1980) may also be reported. Beta can be useful when items are highly heterogeneous or when the goal is to focus on the worst-case scenario of reliability. It is particularly valuable when item intercorrelations vary widely, as it provides a lower bound estimate of internal consistency. The decision to include beta should be based on the nature of the scale: beta may be informative when the scale’s items differ in how strongly they load on the latent construct, but it is less relevant when items are more homogeneous (Kalkbrenner, 2023).

#### Composite reliability

Composite reliability, often used in psychometrics, assesses the overall reliability of a scale by evaluating the ratio of true variance to observed variance in the sum score of the items (Raykov et al., 2016). This metric is based on a unit-weighted sum (linear combination) of items, where each item contributes equally to the composite score. Composite reliability is denoted by the coefficient *ρ_Y_* and typically increases with the number of items in the scale, meaning longer tests generally exhibit higher reliability, while shorter tests tend to have lower reliability (Raykov and Marcoulides, 2011).

#### Average interitem correlation

Another precise measure of internal consistency is the average interitem correlation. It is free of item count influence and thus gives a purer estimate of the underlying cohesion among items (Streiner et al., 2015). When constructing and optimizing tests, the emphasis should be on steering toward an optimal mean interitem correlation rather than chasing a defined level of alpha/omega. When evaluating a wide-ranging trait such as the extraversion dimension of personality, an average correlation as modest as 0.15–0.20 might be appropriate; however, when focusing on a more specific characteristic like talkativeness, a higher average correlation, potentially within the 0.40–0.50 bracket, would be required (Clark and Watson, 2019).

#### Test–retest reliability

If longitudinal data have been collected, test–retest reliability should be used to ensure that measurement variation is attributable to replicable differences between individuals regardless of time, target phenomenon, or respondent profile (Aldridge et al., 2017; Polit, 2014). For psychological/psychiatric scales, two suitable methods are the Bland–Altman limits of agreement (Bland and Altman, 1986), which assess agreement between two numeric scores of repeated measurements, and the intraclass correlation coefficients (Shrout and Fleiss, 1979), which quantify the extent to which two or more ratings for each respondent (within-individual) are statistically similar enough to discriminate between respondents. There are ten forms of intraclass correlation coefficients; the choice depends on the study’s specific theoretical and methodological requirements (Koo and Li, 2016).

Do not estimate all of them and then only report the largest; these are usually based on assumptions that do not reflect the intended scenarios (Youngstrom et al., 2019).

#### Agreement and measurement precision

Measurement error, i.e., the discrepancy between the true value of a variable and the observed value due to inaccuracies in the measurement process, can stem from factors like random errors (e.g., instrumentation error, variation in measurements under identical conditions) or systematic errors (e.g., observer bias) (Hernaez, 2015). Two important metrics for understanding this error in psychological scale development are the standard error of measurement (*SE_m_*) and the minimal detectable change.

The *SE_m_* quantifies the expected variability of an individual’s observed scores around their true score due to measurement error across repeated measurements. It is calculated using the standard deviation and the reliability coefficient of the measurement tool, allowing researchers to construct confidence intervals around the true score. This approach provides insight into the precision and reliability of the measurement tool (de Vet et al., 2017) and is crucial for evaluating the precision of measurement tools, particularly in repeated measurements. On the other hand, the minimal detectable change identifies the minimum change necessary to consider a change in score as real rather than due to measurement error. It is typically calculated using the *SE_m_* and a chosen confidence level (e.g., 1.96 for 95% confidence). This metric is crucial in clinical and research settings for reliably detecting meaningful changes (Geerinck et al., 2019). When combined, *SE_m_* and minimal detectable change provide insights into the reliability and stability of a measurement tool. They delineate the range within which the true score may lie and specify the magnitude of score changes necessary to confirm that the observed change is statistically and clinically significant (de Vet and Terwee, 2010).

Additionally, limits of agreement (Bland and Altman, 1986) describe the range within which the differences between two measurement methods for the same subject are expected to fall. This approach should be especially used to assess the agreement between different measurement techniques.

Another useful measure is the coefficient of variation, which expresses the standard deviation as a percentage of the mean score, providing a normalized indicator of score dispersion. A lower coefficient of variation reflects less relative variability and more precise measurements, making it especially useful when comparing variability across different scales or units with ratio scales that have a true zero point (Riemann and Lininger, 2018). However, caution should be exercised when using coefficient of variation with ordinal data or scales without a true zero, as it can be misleading.

For the analysis of continuous scores, *SE_m_*, minimal detectable change, limits of agreement, and coefficient of variation offer robust insights into the measurement precision and the ability of the measurement tool to detect meaningful changes. For categorical or ordinal data, specific agreement measures, such as Cohen’s kappa or the intraclass correlation coefficient (ICC), aids assess the consistency of classification outcomes. These measures are crucial in evaluating the reliability of diagnostic or classification tools (Mokkink et al., 2020; Swan et al., 2023).

Importantly, addressing and minimizing measurement error is particularly critical when adapting scales for diverse populations because systematic errors—such as biases in response styles, cultural misinterpretations, or biases at the construct, method, or item level—can obscure true similarities or differences across groups and inflate observed variability (Boer et al., 2018).

### Step 16: Test the validity

The validity of a scale is assessed mainly through four key approaches: content, criterion-related, convergent, and discriminant validities (DeVellis and Thorpe, 2022; Raykov and Marcoulides, 2011; Streiner et al., 2015). Each of them is associated with various subcategories and aspects.

#### Content validity

Content validity examines the extent to which the scale items represent and are relevant to all aspects of the targeted construct (Almanasreh et al., 2019; Haynes et al., 1995). It ensures that the scale’s items thoroughly cover the content domain associated with the construct. It is mainly assessed through evaluation by subject matter experts (step 7) and target population representatives (step 9).

#### Criterion-related validity

Criterion-related validity is the extent of relationship (usually squared multiple correlation) of a scale score to an external criterion measure (i.e., the score of a validated measurement instrument or an accepted “gold standard”). It includes both, where the scale predicts future outcomes, and concurrent validity, where the scale correlates with a criterion measured at the same time. Notably, a theoretical rationale for the association between the scale score and the criterion is not mandatory (DeVellis and Thorpe, 2022). The criterion-related validity is primarily of practical interest, it focuses on the strength of the empirical relationship between the measure and the criterion rather than on the comprehension of the underlying processes.

#### Construct validity

Construct validity refers to how well an inventory measures the theoretical construct it is intended to measure. It encompasses theoretical assumptions underlying the instrument (McDonald, 1999) and involves the demonstration that the measure not only captures the essence of the intended construct but also aligns with the theoretical underpinnings of that construct (DeVellis and Thorpe, 2022). Construct validity requires both convergent and discriminant validity (Boateng et al., 2018; Nestor and Schutt, 2019).

#### Convergent validity

Convergent validity involves validating both the measure of a psychological or psychiatric construct and the underlying theory of the construct itself (Strauss and Smith, 2009). It is typically established by correlating the scale score with validated measures of the same or related constructs (Westen and Rosenthal, 2003). The goal is to demonstrate that the scale is associated with these variables in a manner consistent with theoretical predictions. This form of validity goes beyond mere surface similarity, delving into the theoretical underpinnings of the constructs, and ensuring that the scale not only measures what it purports to, but does so in a manner consistent with established theories. Convergent validity is not just about high (but not overly high) correlations, but also about the meaningfulness and appropriateness of these correlations in the context of the underlying theory.

#### Discriminant validity

Discriminant validity, also known as divergent validity, is the degree to which a measure does not correlate or correlate to a low extent with other constructs from which it is theoretically unrelated (Campbell and Fiske, 1959). It also serves as a check against the redundancy of the new measure, ensuring that it captures a unique aspect of a construct rather than merely replicating existing measures (Messick, 1995). For instance, if a new scale is intended to measure anxiety, but it highly correlates with an intelligence test, it may suggest issues with scale construction or underlying theoretical assumptions (Streiner et al., 2015).

#### Evaluating association strengths

The strength of associations in correlations and standardized regressions is often categorized using the following ranges: A very weak relationship is typically indicated by values between 0.00 and 0.19, while a weak relationship falls between 0.20 and 0.39. Moderate relationships correspond to values from 0.40 to 0.59, strong relationships are represented by values from 0.60 to 0.79, and very strong relationships are indicated by values between 0.80 and 1.00 (Campbell, 2021). It is important to note, however, that these classifications are somewhat subjective and may vary depending on the specific context (Campbell, 2021).

## Finalization phase

### Step 17: Revise the item sequencing

The sequencing of the items should be revised based on their factor loadings. To prevent important items from being overlooked due to respondent fatigue (Sudman et al., 1996), the item with the highest factor loading should be positioned at the start of the scale. When scales include both positive and negative items, such as the in-Session Patient Affective Reactions Questionnaire (Stefana et al., 2023, 2024b), the item with the highest loading in each category should be prioritized at the beginning of the scale (Wilson, 2023). Specifically, it is advisable to begin with the “positive” item with the highest factor loading, followed by the “negative” item with the highest loading. Likewise, for multidimensional scales, the items with the highest factor loading in the respective subscale/dimension should be given precedence at the beginning of the scale. The remaining items can then follow in a random order. This strategic placement facilitates more accurate responses and improves the scale’s internal structure.

### Step 18: Prepare an inventory manual and/or the anchor article

The ultimate step in developing a new measurement instrument involves disseminating it to a broader audience. A concise yet comprehensive manual should be created, including essential components such as the theoretical foundations of the instrument, detailed procedures for administration, scoring, and interpretation of results, along with documentation of its psychometric properties (DeVellis and Thorpe, 2022; Streiner et al., 2015). Importantly, selective reporting should be avoided by including all measures used in the analyses and interpreting results based on both data and theoretical rationale. This manual should also provide clear guidelines for norming the instrument and address any special administration rules. To enhance the tool’s accessibility and impact, developers should submit it for classification by relevant regulatory bodies and ensure it is indexed in test repositories (Dillman et al., 2014; Irwing et al., 2018), such as the Health and Psychosocial Instruments (HaPI) database (https://www.bmdshapi.com/) or the Buros Center for Testing (https://buros.org/). Additionally, sharing the manual on open-access platforms like the Open Science Framework (OSF; https://osf.io) can further extend its reach. Publishing the research supporting the development and validation of the instrument in peer-reviewed journals is also advisable to ensure transparency and credibility (Wilson, 2023).

Importantly, periodic revisions are recommended to account for advances in theory, changes in the construct being measured, or the presence of outdated items. The frequency of these revisions should be guided by empirical testing and feedback from the field, ensuring the instrument remains relevant and reliable over time (McCoach et al., 2013; Zickar, 2020). Changes in technology are also making it possible to combine traditional scales with information such as meta-data about response time, eye tracking while completing tasks, as well as entirely different sources of information such as performance tasks, geolocation, passive data from smart devices, implicit association tests, and many more modalities (Dillman et al., 2014; Youngstrom et al., 2017).

## Conclusion

Developing and validating a scale is a complex, multistep process that demands both methodological rigor and flexibility. This article provides an overview of this process (Table 1) to improve accessibility to and transparency in scale development. However, it is important to note that the sophistication of scale development, which can vary across studies, means that this article serves as an introductory guide rather than a comprehensive manual. Although our focus has been on psychological and psychiatric scales, the principles and guidelines outlined are largely transferable to the development of measures across the health, behavioral, social, and educational domains.
